# Caregiver Version Shared Decision-Making Questionnaire for Adult Child and Parents with Dementia: A Delphi Study

**DOI:** 10.1093/geroni/igaf122.2974

**Published:** 2025-12-31

**Authors:** Liu Huanran, Vivian Lou

**Affiliations:** The University of Hong Kong, Hong Kong, China (People’s Republic); The University of Hong Kong, Hong Kong, China (People’s Republic)

## Abstract

Adult children constitute the largest group of family caregivers globally and play a pivotal role as primary decision-makers and long-term caregivers for parents with dementia. While shared decision-making (SDM) traditionally involves patients and physicians in clinical settings, dementia care often extends beyond formal healthcare, with decisions about care arrangements, daily care, medication, and finances frequently made by people with dementia and adult child caregivers. Despite the importance of SDM in dementia care, existing research has primarily focused on physician-patient dynamics, leaving a significant gap in understanding patient-caregiver SDM. To address this gap, we adapted the Shared Decision-Making Questionnaire (SDM-Q) into an adult-child caregiver version and evaluated its content validity through a two-round online Delphi study. This adapted tool aims to assess caregivers’ perspectives on decision-making with their parents. After translation and a focus group with seven caregivers, nine experts (three researchers, four practitioners, and two caregivers) evaluated the scale’s importance, relevance, and clarity using a nine-point Likert scale. Changes, such as replacing “patient” with “my father/mother,” were made based on expert feedback to reduce stereotypes. In the first round, item-level content validity indices (I-CVI) ranged from 0.78 to 1 across domains, with Ave-CVI scores of 0.93 (importance), 0.94 (relevance), and 0.89 (clarity). All items achieved moderate to high consensus. In the second round, I-CVI and Ave-CVI improved to 1, indicating excellent content validity and high expert consensus. These results suggest the SDM-Q-Carer is a valid tool for exploring decision-making involvement among adult child caregivers, though further validation is recommended.

